# Socioeconomic benefits associated with bats

**DOI:** 10.1186/s13002-024-00720-w

**Published:** 2024-08-20

**Authors:** Siya Aggrey, Innocent B. Rwego, Eric Sande, Joyce D. Khayiyi, Robert M. Kityo, Charles Masembe, Rebekah C. Kading

**Affiliations:** 1 Department of Zoology, Entomology and Fisheries Sciences, College of Natural Sciences, Makerere University, P. O. Box 7062, Kampala, Uganda; 2 Uganda Wildlife Research and Training Institute, P. O. Box 173, Kasese, Uganda; 3 Department of Biosecurity, Ecosystems and Veterinary Public Health, College of Veterinary Medicine Animal Resources and Biosecurity, Makerere University, P. O. Box 7062, Kampala, Uganda; 4 Department of Microbiology, Immunology, and Pathology, Center for Vector-Borne Infectious Diseases, Colorado State University, Fort Collins, CO USA

**Keywords:** Bats, Conservation, Welfare, Economics, Indigenous knowledge, Traditional knowledge, Benefits, Social ecosystem

## Abstract

**Background:**

While bats are tremendously important to global ecosystems, they have been and continue to be threatened by loss of habitat, food, or roosts, pollution, bat diseases, hunting and killing. Some bat species have also been implicated in the transmission of infectious disease agents to humans. While One Health efforts have been ramped up recently to educate and protect human and bat health, such initiatives have been limited by lack of adequate data on the pathways to ensure their support. For instance, data on the role of bats in supporting different components of human welfare assets would be utilized as a stepping stone to champion conservation campaigns. Unfortunately, these data are limited and efforts to synthesize existing literature have majorly focused on few components human welfare leaving other important aspects.

**Methods:**

Here, we analyze benefits associated with bats in the context of welfare economics considering all the asset components. We surveyed scientific and gray literature platforms utilizing particular keywords. We then classified these values using integrated approaches to understand different values across human welfare assets of “health,” “material and immaterial assets,” “security or safety” and “social or cultural or spiritual relations”.

**Results:**

We found 235 papers from different countries indicating that bats play fundamental roles in supporting human welfare. These benefits were more prevalent in Asia and Africa. In terms of the use of bats to support welfare assets, bats were majorly utilized to derive material and immaterial benefits (*n* = 115), e.g., food and income. This was followed by their use in addressing health challenges (*n* = 99), e.g., treatment of ailments. There was a similarity in the benefits across different regions and countries.

**Conclusion:**

These results indicate potential opportunities for strengthening bat conservation programs. We recommend more primary studies to enhance understanding of these benefits as well as their effectiveness in deriving the perceived outcomes.

**Supplementary Information:**

The online version contains supplementary material available at 10.1186/s13002-024-00720-w.

## Introduction

Bats are exceptionally diverse with approximately 1420 species worldwide [1, 2]. As such, they are cosmopolitan and occupy many ecological niches which include consumption of insects [2, 3], fruits [4, 5], nectar [6], blood and vertebrate animals [7, 8], and generation of nutrient-rich guano [9, 10]. These services all carry some intrinsic value to human populations living in association with bats, which can be measured, considering the appropriate socioeconomic context within the social ecosystem. While these mammals have been implicated for a range of disease occurrences, the socioeconomic benefits associated with them are critical in sustaining different constituents of human well-being, i.e., security; material and immaterial assets; health; and social, spiritual and cultural relations. This includes influencing the general capabilities of the people, i.e., in terms of what they can do and be in their life. This can be in terms of opportunities for knowledge access, enjoying supportive relationships, cultures among others. Because a person’s capability set is highly context dependent, i.e., varies with environmental, social and economic context, the benefits associated with bats across well-being can as well be highly variable [11]. Therefore, conservation interventions targeting bat populations can be better designed with availability of robust data on the benefits (in terms of human livelihoods) associated with them.

While there are many examples of socioeconomic benefits associated with bats, systematic assessments of these values across different components of human welfare assets are rare. Additionally, assessments that consider all the socioeconomic components of health, safety, material and immaterial assets as well as sociocultural relations are yet to be conducted. Mickleburg and colleagues analyzed the global bat meat consumption revealing the widespread benefit of bats as food [12]. Similarly, recent assessments by Tanalgo and colleagues revealed widespread consumption of bats as food with implications on conservation [13]. These are critical results for strategic design of conservation interventions. However, inclusion of other components of the socioeconomics that are critical in the social ecosystem as exemplified in the cascade framework for ecosystems services assessment would provide holistic interventions for conservation programming [14]. Socioeconomic benefits are often context dependent, i.e., human livelihood may vary across space and time, and the latest benefits associated with bats would be crucial in supporting conservation programming [11, 15]. Owing to the context dependence of human well-being, existing literature ought to be updated to support current policy changes [15]. Recently, Tackett and colleagues attempted to analyze the use of bats for medicinal purposes globally [16]. Whereas they attempted to include both latest scientific and gray literature, their focus was only on the medicinal benefits attached to bats ignoring other components of the socioeconomy, i.e., safety and security, material and immaterial factors, social, spiritual and cultural relations. Another study by Low and colleagues studied the cultural benefits associated with bats in the Asia pacific region [17]. This was an important milestone in documenting cultural benefits that form an important component of human livelihood and yet are often ignored. It also provides insights on the benefits of bats in other regions and consequently supports conservation programming. However, their major focus on the Asia region provides results that may not be applicable to other regions. This is because of the context dependency of human owing to different factors across, i.e., economic, environmental and social aspects [11, 15]. Therefore, using such results to support bat conservation programming in other areas may not yield significant outcomes.

Besides socioeconomic aspects, Ramírez-Fráncel and colleagues analyzed ecological values associated with bats [18]. This was an important step in documenting ecosystem values of bats across different scales with potential of supporting bat conservation planning. However, their analysis did not include other aspects of the human welfare economies that would be crucial in understanding the social ecosystem with wider consequences on conservation interventions [14]. Notably, they did not include social, cultural and spiritual relations as well as safety and security aspects. Additionally, because of the context dependence of socioeconomic values, the results of this study may not be useful in supporting bat conservation interventions in other locations [19]. There is thus need for an updated and explicit review of socioeconomic benefits of bats across different areas. This will provide an opportunity for promoting bat conservation across different scales. Considering the fact that community needs and cultural interactions with bats are highly diverse, updated and explicit data on socioeconomics are critical. Here, we address this gap in the literature with an analysis of the socioeconomic benefits of bats across different continents.

Given the wide-ranging socioeconomic benefits of bats, an analysis that integrates different components of human welfare while allowing for their comparison would be very useful. Existing ecological frameworks like socioeconomic impact classification, i.e., “health,” “material and immaterial,” “safety and security” and “social, cultural and spiritual relations,” would provide useful insights regarding the benefits of bats across human welfare [20]. It would also enable the identification of regions with higher benefits attached to bats, potentially informing management interventions to protect human health and livelihoods. While other globally focused reviews, e.g., [12, 21, 22], provided useful insights regarding some aspects of socioeconomic benefits of bats, use of a fairly explicit framework like the “socioeconomic impact classification” allows for explicit analysis of different components of socioeconomic benefits generated by bats. These existing reviews thus ought to be enhanced with updated analysis of the benefits of bats across different components of human welfare. The use of utilitarian approaches of monetizing the benefits of bats as used in other studies, e.g., [11, 23–25] seems an obvious route for quantifying socioeconomic benefits. Yet it is unlikely that monetizing benefits will provide a useful basis for comparison because converting all benefits into monetary values is difficult, if not impossible. To capture the full socioeconomic benefits of bats, dimensions that go beyond monetary values ought to be considered.

In this study, we adopted an existing framework that focused majorly on the socioeconomic impacts of invasive alien species [20]. We modified this approach to fit bats by only focusing on the positive benefits which are critical in supporting conservation outreach and awareness programs. This framework was adopted to provide a standardized method to categorize the broad range of socioeconomic benefits associated with bats. This approach uses human activities that result from the benefits attached to bats as a common metric for assessing the magnitude of the benefits. In so doing, it enables direct comparisons across regions regarding the magnitude of the benefits attached to bats. Additionally, as it has been widely recognized that values and benefits are different within the social ecosystem [26], this study integrated the ecosystem services framework to delineate different factors. To our knowledge, this study provides the first explicit large-scale (beyond regional level) assessment of the socioeconomic benefits associated with bats. Additionally, it is the first study to simultaneously provide an analysis of different components of human welfare (i.e., “health,” “material and immaterial assets,” “security and safety,” “social and spiritual relations”) supported by bats. Other existing studies only included a few components of human welfare while others mainly focused on ecosystem services. By undertaking this assessment, we aim to further our understanding of the global human–bat interactions and to identify knowledge gaps directing future studies on assessment of benefits of bats.

## Methodology

### Literature search

We conducted a literature review, searching the databases PubMed, Web of Science, Google Scholar and Scopus on the socioeconomic values of bats. The search was limited to English terms and utilized Boolean operations including “AND” and “OR” with a combination of the following key terms: bats, flying fox, values, importance, benefits, and Chiropteran. Some of the search terms included; (TITLE (bat) AND TITLE (values) OR TITLE (benefits)) and (TITLE ( flying AND fox) AND TITLE (values) OR TITLE (benefits)), (TITLE ( chiroptera) AND TITLE ( values) OR TITLE ( importance)). The reference sections of these studies were also scanned to identify additional studies worthy of inclusion. As some socioeconomic benefits are underreported, scientific literature was complemented with gray literature search. Our last search was conducted in June 2023, and there was no restriction on the year of publication of the article. After the initial search, duplicates were removed manually. The remaining articles were screened by title and abstract.

### Inclusion and exclusion criteria

Articles were included in the review if they reported socioeconomic values of bats across human well-being components of health, safety, material, and immaterial assets as well as social, cultural, and spiritual relations. The subcomponents and examples across these assets that were considered are listed in Table 1. This search was performed across the world, and ecological values were not included as it does not directly form part of human well-being. Any literature that did not satisfy the inclusion criteria was excluded from the review. These studies were screened by two authors to improve the quality checks. In the first place, one author screened titles and a second screening was performed by another on the abstracts. The roles were switched between the two authors, and discrepant results were discussed to make a decision.

**Table 1:** Human well-being categories including some specific examples of societal benefits of bats

Human well-being constituent supported by bats	Subcategories of human well-being	Examples of activities undertaken to ensure livelihood (with respect to bats)
Material and immaterial assets	Adequate livelihoods Sufficient nutritious food Shelter Access to goods	Bats are eaten as food Bat guano is collected and used as fertilizer for crop production Bats reduce pests for crops
Safety	Personal safety Secure resource access Security from disasters	Bats alert in case of an imminent danger to human communities
Health	Strength Feeling well Access to clean air and water	Bats are used to treat health complications Bats consume pests that cause diseases
Social, spiritual, and cultural relations	Social, spiritual, and cultural practice Mutual respect Friendship	Bats are treated as gods, totems and enhance relationships

### Data extraction and analysis

Data were extracted following a summary protocol that had the link to the publication, nature of the article (peer reviewed/gray), year of publication, title, country, region, continent and description of the benefit. We classified values across different well-being components including health, safety, material and immaterial assets, social, cultural and spiritual relations. Well-being in this study was defined using the capability approach [11]. Examples of the well-being subcomponents are listed in Table 1. For each article, incidences of mention of a benefit related with that in Table 1 were noted. Some articles that noted these benefits across different subcomponents were also noted. As scientific literature on socioeconomic benefits of bats is scanty, we included data from other review papers to complement our data. In incidences where a review paper reported a benefit that is linked to a reference already included, it was not taken into consideration. This was done to avoid duplicate results. Attempts were made to score the magnitude of the socioeconomic benefits; however, it was impractical because existing studies and reports did not report how absence of bats could influence activities undertaken by human communities. Therefore, this study only stopped with classifying the benefits. This classification of benefits was done in excel with each record scored. Analysis of data only focused on grouping different benefits across different scales, e.g., continents and regions. These data were then analyzed through aggregating the scores using $highcharter$ package [21] in R version 4.3.1 [27]. The Sankey diagrams produced would then represent the commonness of the benefits across different regions as well as the intensity.

## Results

A total of 2,610 titles were identified from the database searches. An additional 32 articles were identified through other means (including bibliographical search), leading to a total of 2,642 studies. After screening these items to determine whether the inclusion criteria were met, and removing duplicates, a total of 253 articles were assessed for eligibility. The rest of the articles did not have contents for the values but were rather focused on other aspects. Additionally, some of them were duplicates. Of these, an additional 18 were excluded after further screening for whether they contained aspects of socioeconomic benefits. This resulted in a total (n) of 235 studies that were included in our analysis.

### Values of bats across regions and countries

**Fig. 1:**
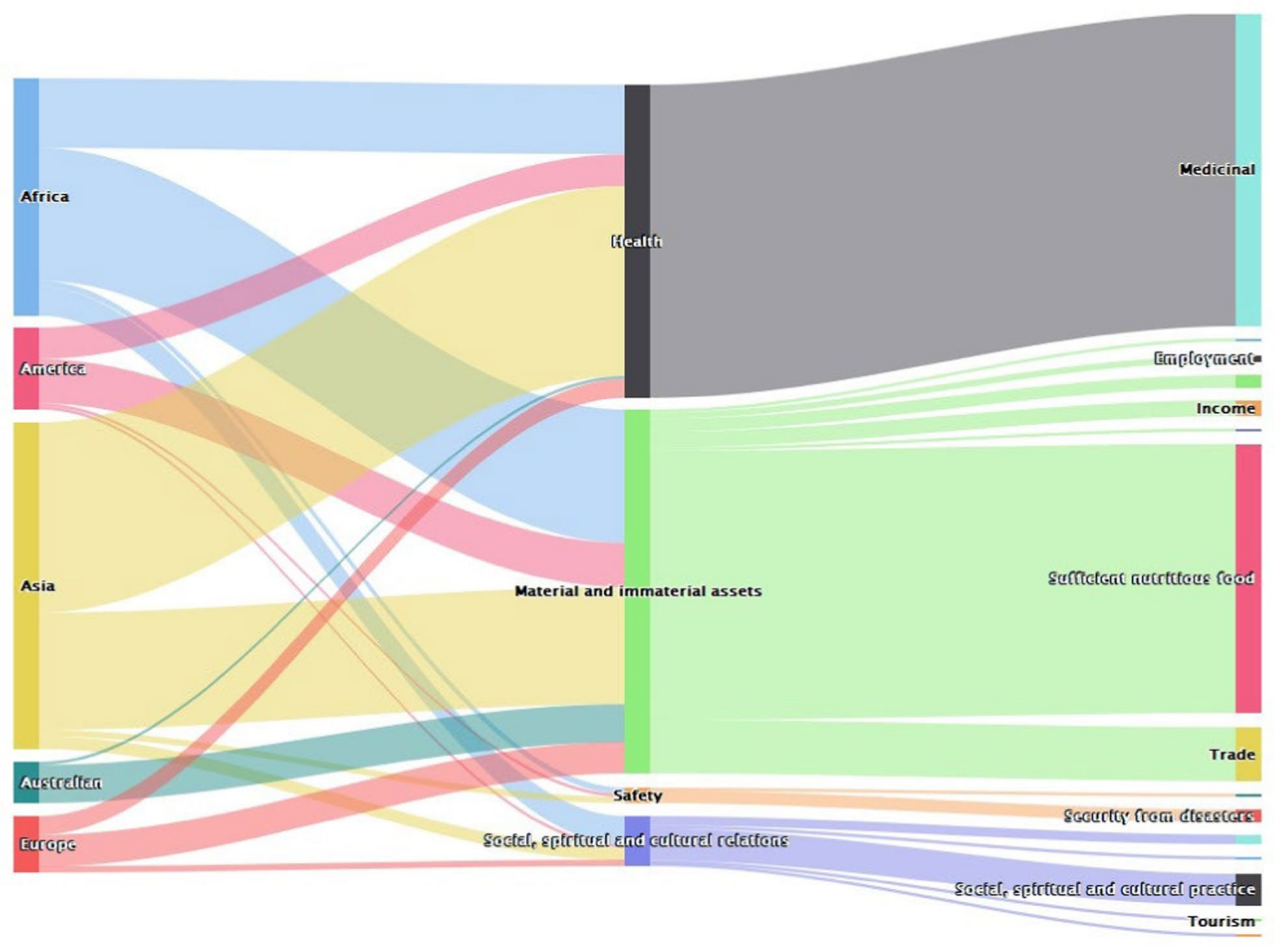
Reported values of bats across different continents (*n* = 235)

A summary of the results from all 235 papers included in the analysis is presented by geographic region (Fig. 1). Each of the different categories of socioeconomic benefits of bats was represented across different geographic areas, although to different extents. It is unclear whether these results reflect differences in reporting and publishing practices, in different regions or different types of activities in each of these categories. The types of activities in each socioeconomic category that were reported from different regions are discussed further below.

**Fig. 2:**
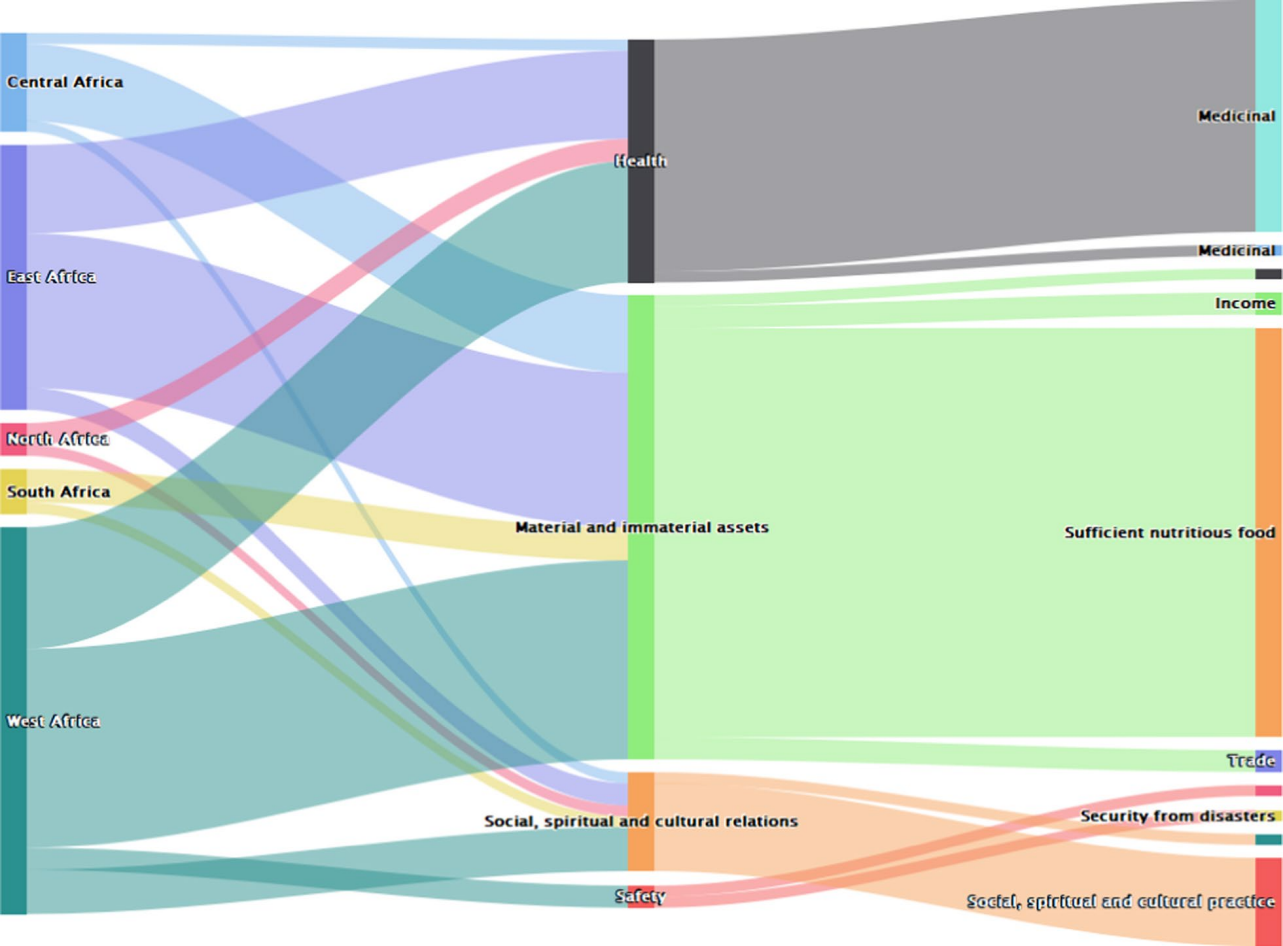
Values of bats across different regions of Africa (*n* = 75)

Within Africa, records indicate bats were valuable especially in West and East African regions (Fig. 2). They were highly valued for their benefit in form of material and immaterial assets. Notably, sufficient, and nutritious food, income and trade benefits were derived from bats (Fig. 2).

**Fig. 3:**
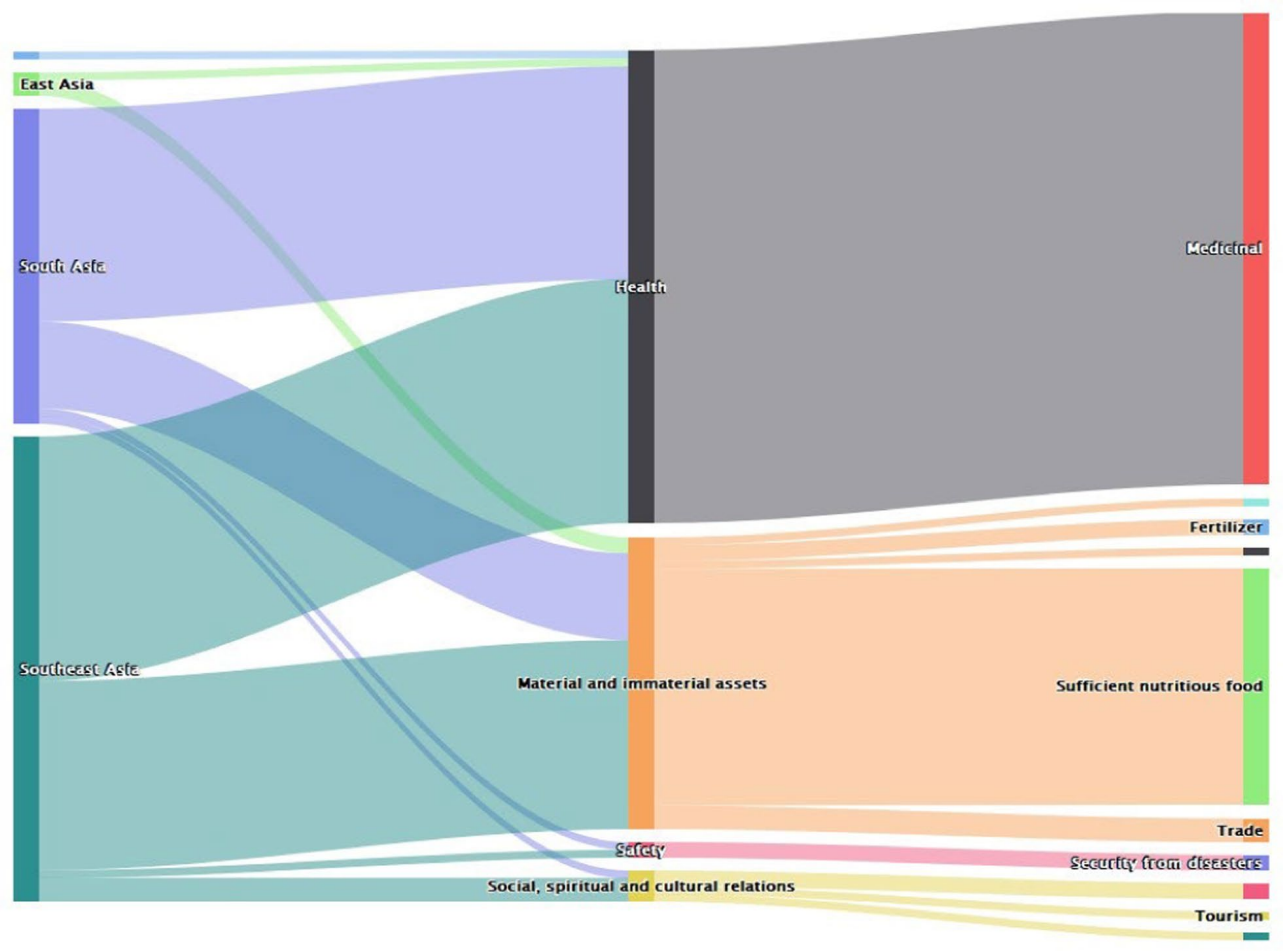
Values of bats in Asia (*n* = 103)

In Asia, data indicate bats influenced human livelihoods and well-being more in the South and Southeast areas. They were majorly valued for health aspects of human welfare. The other important value was on the material and immaterial assets which culminated into sufficient and nutritious food (Fig. 3).

**Fig. 4:**
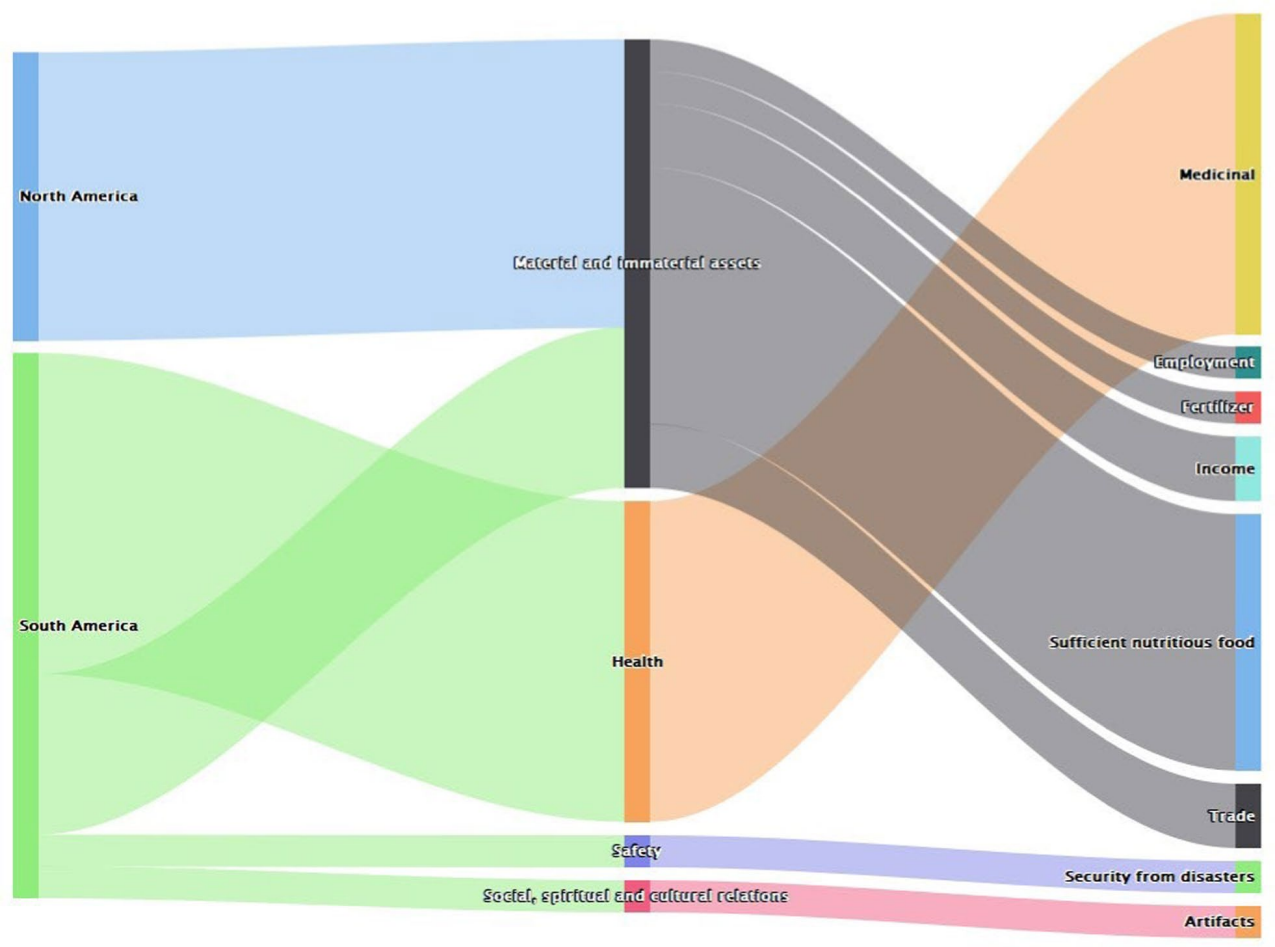
Values of bats in the Americas (*n* = 26)

In the Americas, studies indicate values of bats in the South American region were more than that in the North American region (Fig. 4). In both regions, bats were valued for material and immaterial assets. These material and immaterial assets were in the form of fertilizers (e.g., guano), income, sufficient and nutritious food. Meanwhile, in the South American side, benefits centered around health and human communities utilized them to deliver this value through using it as a medicine (Fig. 4).

**Fig. 5:**
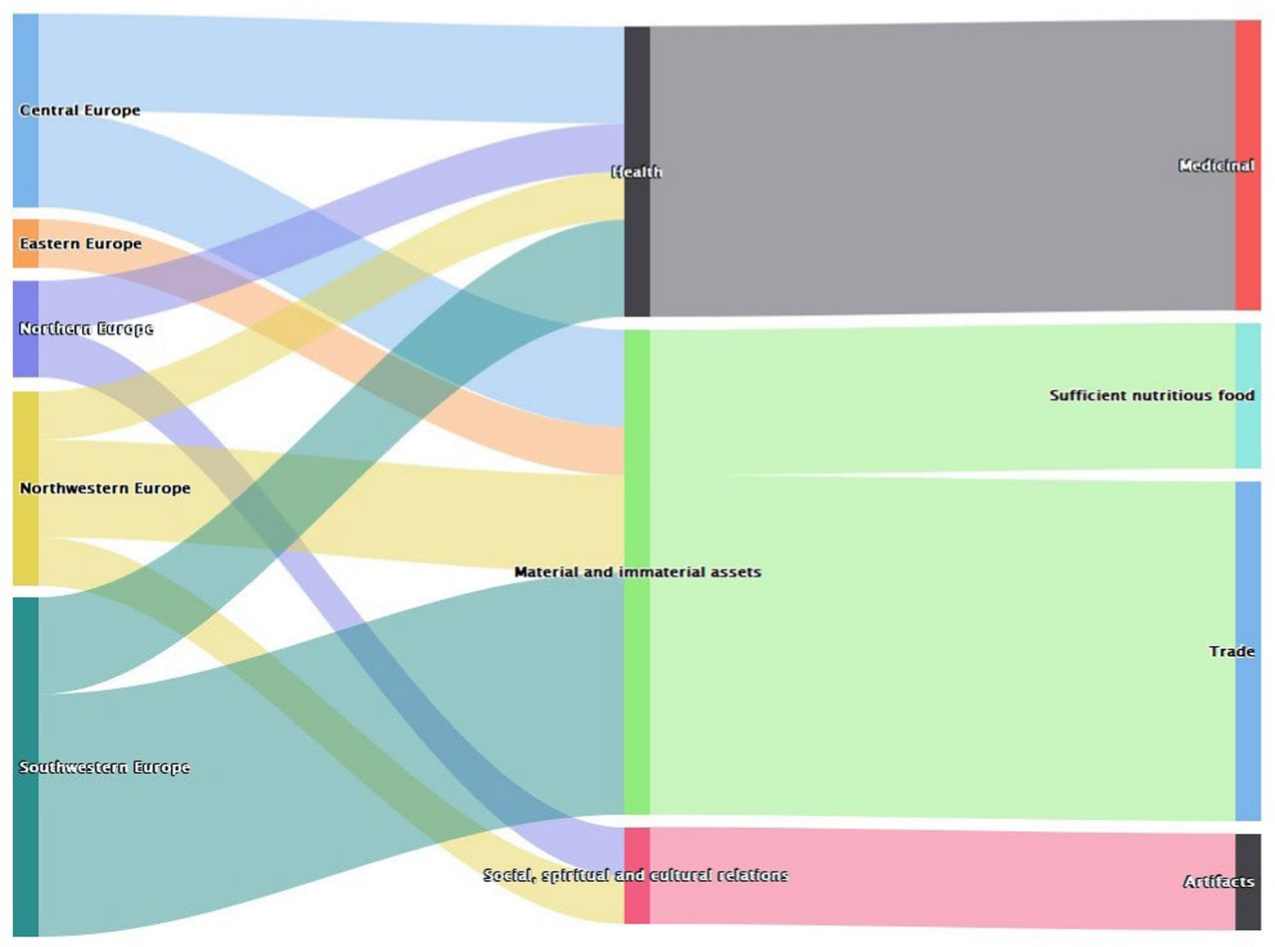
Values of bats in Europe (*n* = 18)

In Europe, records indicate bats were valued majorly in the Southwestern parts (See Supplementary Material 1) and were majorly related with material and immaterial assets (Fig. 5). Through this pathway, it majorly influenced trade and activities to ensure sufficient food. Other benefits included supporting health and were majorly used for medicinal purposes.

**Fig. 6:**
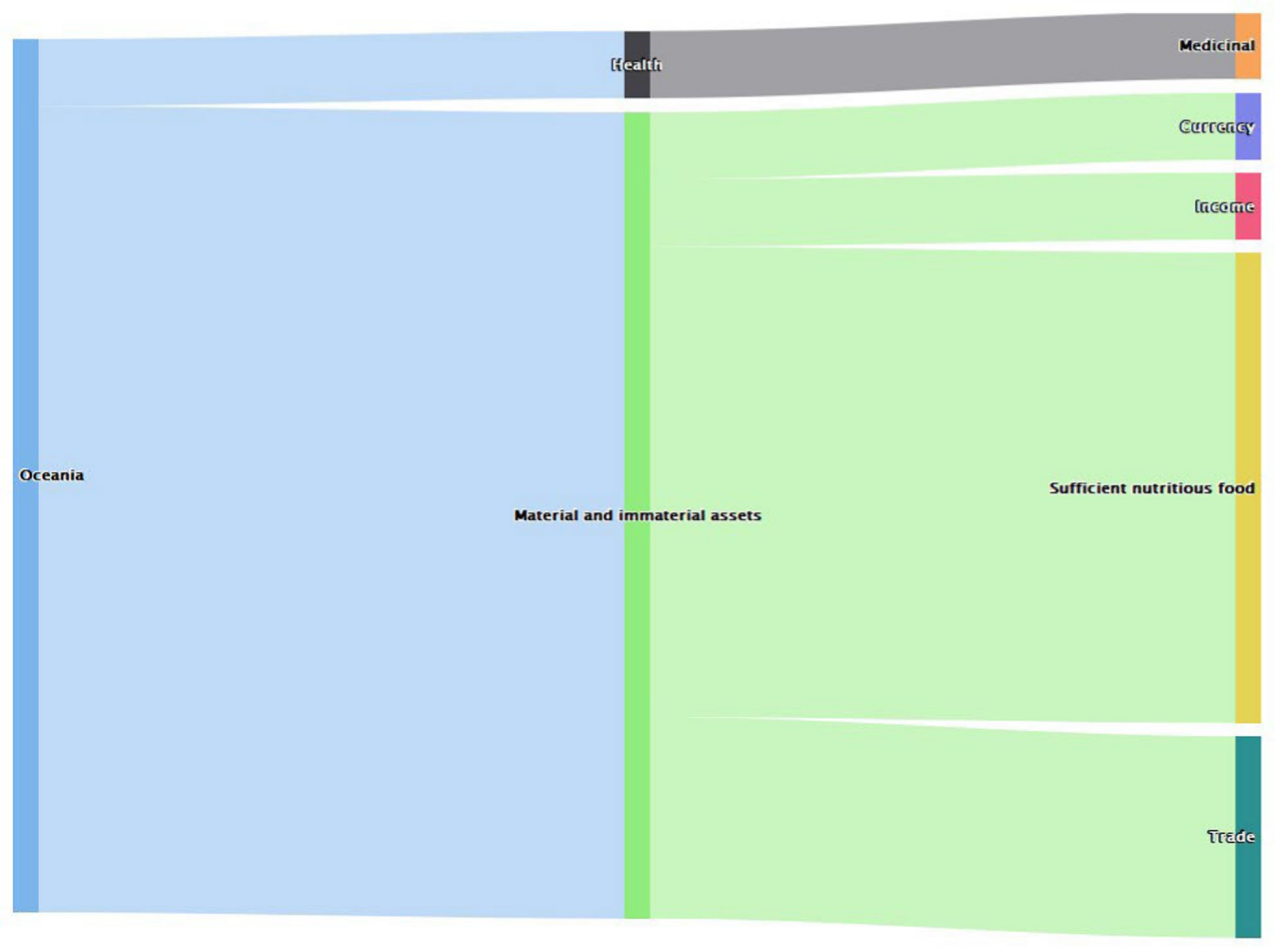
Values of bats in Oceania region (*n* = 13)

In Oceania, studies indicate bats were majorly associated with material and immaterial components of human well-being. It influenced activities that ensure trade, income generation, transactions and food resources (Fig. 6).

### Socioeconomic benefits associated with bats in different areas and across different categories of human welfare

#### Material and immaterial benefits

**Fig. 7:**
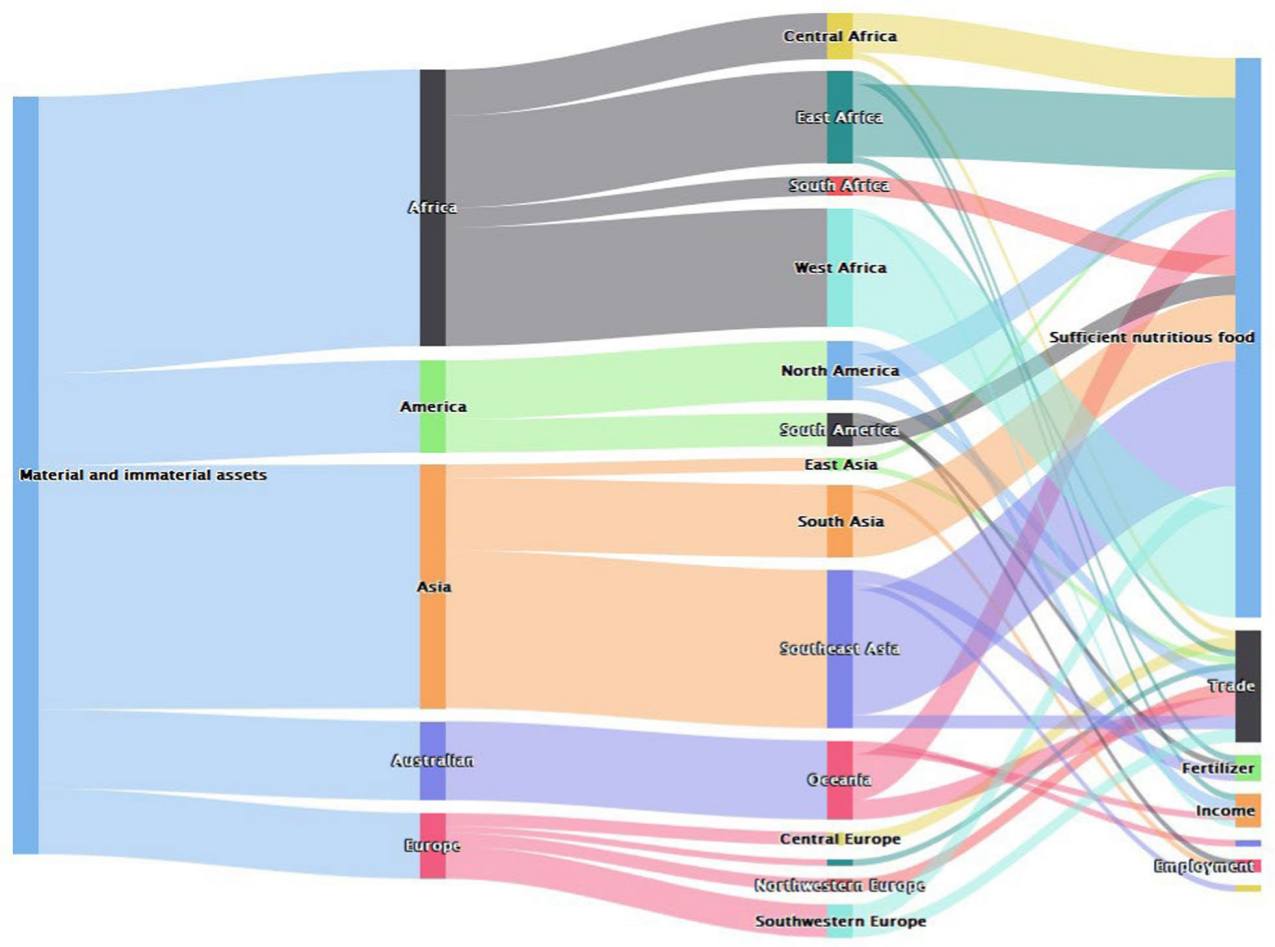
Geographical distribution of papers reporting material and immaterial benefits of bats (*n* = 115)

Material and immaterial benefits associated with bats were reported more in Africa and Asia compared to other regions (Fig. 7). In these two regions (Africa and Asia), material and immaterial assets were primarily reported in form of supporting sufficient and nutritious food. Other benefits attached to bats within this category and in the two regions included supporting trade, providing fertilizers and income generation (Fig. 7).

These values reflect the critical role that bats play in ensuring resource access by human populations. These resources (material and immaterial) are critical in sustaining human livelihoods and well-being across different scales. In Africa, consumption of bats has been recorded in west African countries including Nigeria, Ghana, Benin, Sierra Leone, Cameroon and Côte D’ivoire. In Nigeria, bats form an important source of protein across different communities [22, 28]. Here bats are hunted and consumed. Similar results have been observed in Cameroon [29], Ghana [24, 30–33], Guinea [22], Côte d’Ivoire [34] and Sierra Leone [35]. Besides hunting of bats for direct benefits including meat, bats were associated with specific ecological services such as enhancing crop production, providing guano as a fertilizer and also suppressing pests. In terms of cash crops, records in Benin and Cameroon indicate bats have significant contribution to enhancing yields of cacao trees through suppressing pests [25, 36]. Bats have also been indicated to be an important source of guano which is used to enhance crop production which is an important ecosystem service [25]. Other material and immaterial benefits associated with bats included generating income through trading activities as observed in Ghana [37].

In Eastern Africa region, bats were reported to be an important source of food, income, guano among others. For instance, anecdotal records from Tanzania and Uganda indicate bat guano to be a valuable resource in ensuring agricultural production. Similar observations were made in Kenya [38]. Besides acting as an important source of guano, bats are hunted and consumed in this region [38, 39]. Meanwhile, in terms of food, bats are an important source of food in form of bush meat within this region [40, 41]. Besides supporting food systems, bats are an important source of income. For instance, in Kenya, bats form an important component of tourism enhancing the welfare of those engaged [38]. Similarly, anecdotal records indicate Python cave (located in Queen Elizabeth National Park in Uganda) to be an important destination for tourists. The tourists often visit this cave to view bats [42]. This generates income not only to those who guide tourists to this site but also contributes to the national revenue. In Tanzania, bats have been noted to form an important part of E-commerce. Notably, bats are turned into souvenirs and sold globally generating income for those engaged in the business and revenue for the country. While we did not find sufficient evidence for the legal status of this business, it appears to be illegal. Consequently, it has potential negative consequences on bat population health as well as public health [43].

In the southern Africa region, the benefit of bats in terms of material and immaterial assets is prominent especially in South Africa and Madagascar. In this region, bats have been noted to act as an important food resource as well as limiting pests. In Madagascar, bats are hunted and consumed as an important source of protein [44–47]. Meanwhile, in South Africa, bats play fundamental roles in enhancing agricultural production through limiting pest populations. This has been noted in macadamia fields where bats have significantly reduced pests enhancing production [48]. Although scientific reports were not obtained regarding the use of guano in this region, anecdotal records indicate use of bat guano in Mozambique, Zimbabwe, Zambia and Malawi. In Northern Africa, records on the benefits of bats are scanty. However, anecdotal records indicate bats to act as an important source of protein. They are hunted and prepared as a meal.

In Asian countries, bats are majorly valued (in terms of material and immaterial assets) as an important source of food. Other benefits include acting as an important source of revenue, guano, as well as sustaining food production through pollination. In India, bats are hunted and form an important source of protein [49]. Similar observations have been made in Bangladesh [50], Indonesia [51], Malaysia [52], Philippines [53, 54] and Thailand among others [55]. Other benefits include acting as an important source of income and revenue through trade and tourism. Regarding trade, records have been made in Viet Nam where bats get dried and stuck together forming souvenirs [28]. These souvenirs are then sold generating income to those who engage in the business. Meanwhile, in China and Indonesia, bats form an important part of e-commerce [43].

In the Oceania region, material and immaterial benefits have been recorded in the Island nations including Palau, Solomon, and Samoa. In these islands, bats are an important source of food for the local human population. This has been observed in Samoa [56] and Palau islands [30]. The hunted bats are sold generating income. The teeth of bats have also been indicated to be utilized as a form of currency in the Solomon Islands [31]. This is critical in supporting trade enabling access to other needs. In the Oceania region, the bats were majorly associated with sustaining agricultural production through preying on pests. This was indicated in a record by Kolkert and team that indicated bats to contribute significantly to production of cotton [32].

In the Americas, bats have been valued in terms of sustaining agricultural production and generating incomes to those engaged in trade related activities. In terms of agricultural production, Cleveland and colleagues estimated over $700,000 annually as value derived from the pest predation services by Brazilian free-tailed bats [23]. Meanwhile in Brazil, bats have been shown to suppress pests in banana plantations [33]. Similarly, a study by Maslo and colleagues detected DNA of pests in the fecal matter of bats and such pests have a potential to undermine agricultural production [57]. In Chile, bats were shown to limit pest incidences around the vineyards yielding up to $188-$248/ha/year due to bat predation [58]. This reflects the critical role that bats play in sustaining agricultural production. Besides sustaining crop production, bats also generate income for those engaged in trading of souvenirs with bat symbols. This is majorly indicated in anecdotal records. Additionally, in western Brazil, records in the past indicate bats were consumed and formed an important delicacy [36].

In Europe, records indicate bats to be valued largely in terms of sustaining agricultural production as well as generating income and revenue. For instance, in Italy, souvenirs with bat symbols are indicated to be purchased generating income. This has been highlighted on the e-commerce involving bats [43]. Similarly, e-commerce involving bats has been recorded in Germany, UK and Netherlands [43]. In terms of agricultural production, bats have been shown to enhance productivity of livestock in Italy through feeding on pests that would undermine livestock production [59].

#### Health benefits associated with bats

**Fig. 8:**
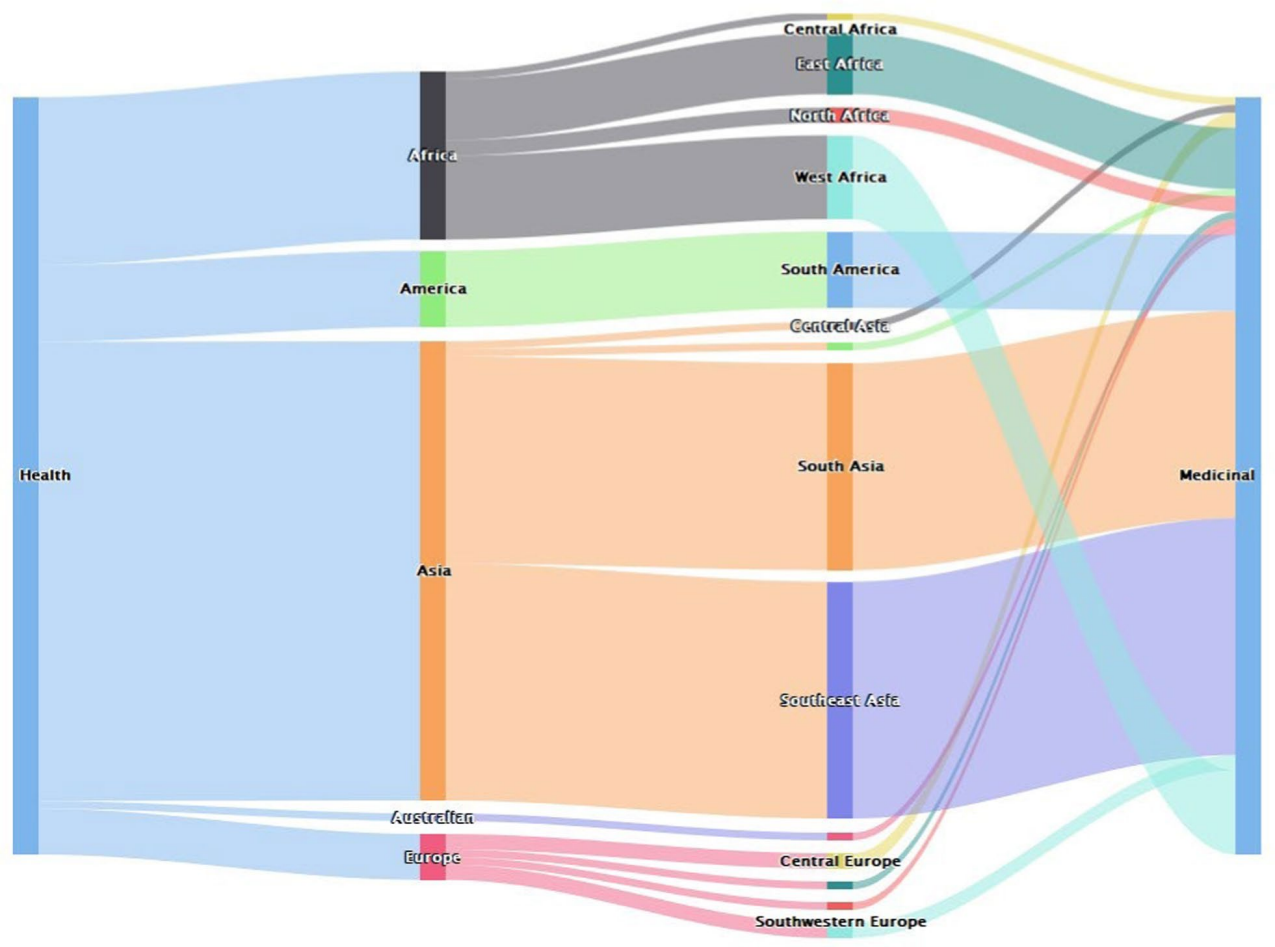
Geographical distribution of papers reporting health benefits of bats (*n* = 99)

This benefit mainly linked with medicinal benefits that addressed several ailments and complications. In Asia, South Asia and Southeast Asia were the regions that had more benefits attached to bats in respect to this livelihood component (Fig. 8).

Throughout the world, bats have directly benefited human communities through providing treatment options for different illnesses. These illnesses include asthma, cough, kidney complications, body aches and infertility. Within Africa, the use of bats for managing health-related issues has been observed in West, Central, East and Southern Africa. In West Africa, bats have been used to treat mental illnesses among individuals in Senegal [60]. The head and sometimes whole body of the bat are prepared and consumed to address such ailments [60]. The perception behind this remedy is bats have night-flying ability inferring a symbol of orientation. Therefore, patients with mental illness accordingly have orientation problems and ingestion of certain bat parts can help them recover from the ailment. Although this record was made a long time ago, it may still be practiced up to date and signifies an important socioeconomic value that community members attach to bats. In communities around south western Nigeria, bats are used to treat abdominal pains and infertility [61]. Similarly in Cameroon, bats have been used to address infertility challenges [40]. Similar records have been made in Benin and Ghana among others. Gray literature also indicates bats are used in Nigeria to control baldness.

In the east African region, particularly Kenya, it has been noted to disperse tree seeds providing opportunities for green environment suitable for human health [38]. Meanwhile, in Uganda, anecdotal records indicate community members in the southwestern part of the country eat bats as they perceive them to increase the body immunity [41]. This community was also noted to associate bat consumption with smooth skin among women. Similarly, young children are fed on bat soup to clear off diseases. At times, a tooth of the bat is tied around the waist of the baby and is perceived to clear off diseases. Meanwhile, in the southern African region, health benefits are majorly reported in Mozambique. These health benefits include treatment of cough, baldness and asthma [62, 63].

In Asian region, bats are associated with treatments for asthma, fever, arthritis, liver diseases, cough and tooth ache, among others. In Bangladesh, it is used to treat asthma, fever and arthritis [64]. Bats are also perceived to enhance immunity of people [64]. Meanwhile, in India, bats are noted to provide zootherapeutic benefits including treatment of asthma, cold, cough, tooth ache and liver diseases [65]. They are also utilized to reduce bed wetting [65]. Similar benefits have been observed within communities in Indonesia, Malaysia, Nepal, Pakistan, Philippines, Pakistan and Thailand [45], [99, 100].

In the Americas, Europe and Australasia, health benefits associated with bats appear to have been common in the past but have declined over time. In countries within the Americas including Brazil, bats are pulverized and used in the treatment of asthma in humans [66]. The pulverized product is also used as a contraceptive for livestock [66]. Additionally, it is also used in reducing addiction to alcohol among humans [66]. Within the USA, bat oil was utilized in the past to treat rheumatism [60]. In Latin America, the vampire bats are noted to have saliva with substances that inhibit blood clotting [60]. These substances have been utilized in preventing clotting of blood in patients [60], [102, 103]. This is crucial for patients with acute ischemic stroke [67], [102]. Meanwhile, in European countries, bats played fundamental roles in providing pharmaceutical products [60]. The use of bats for this benefit seems to have faded over time [60]. In Australia, bats are known to be critical in the treatment of asthma [68].

#### Social, spiritual and cultural benefits associated with bats

**Fig. 9:**
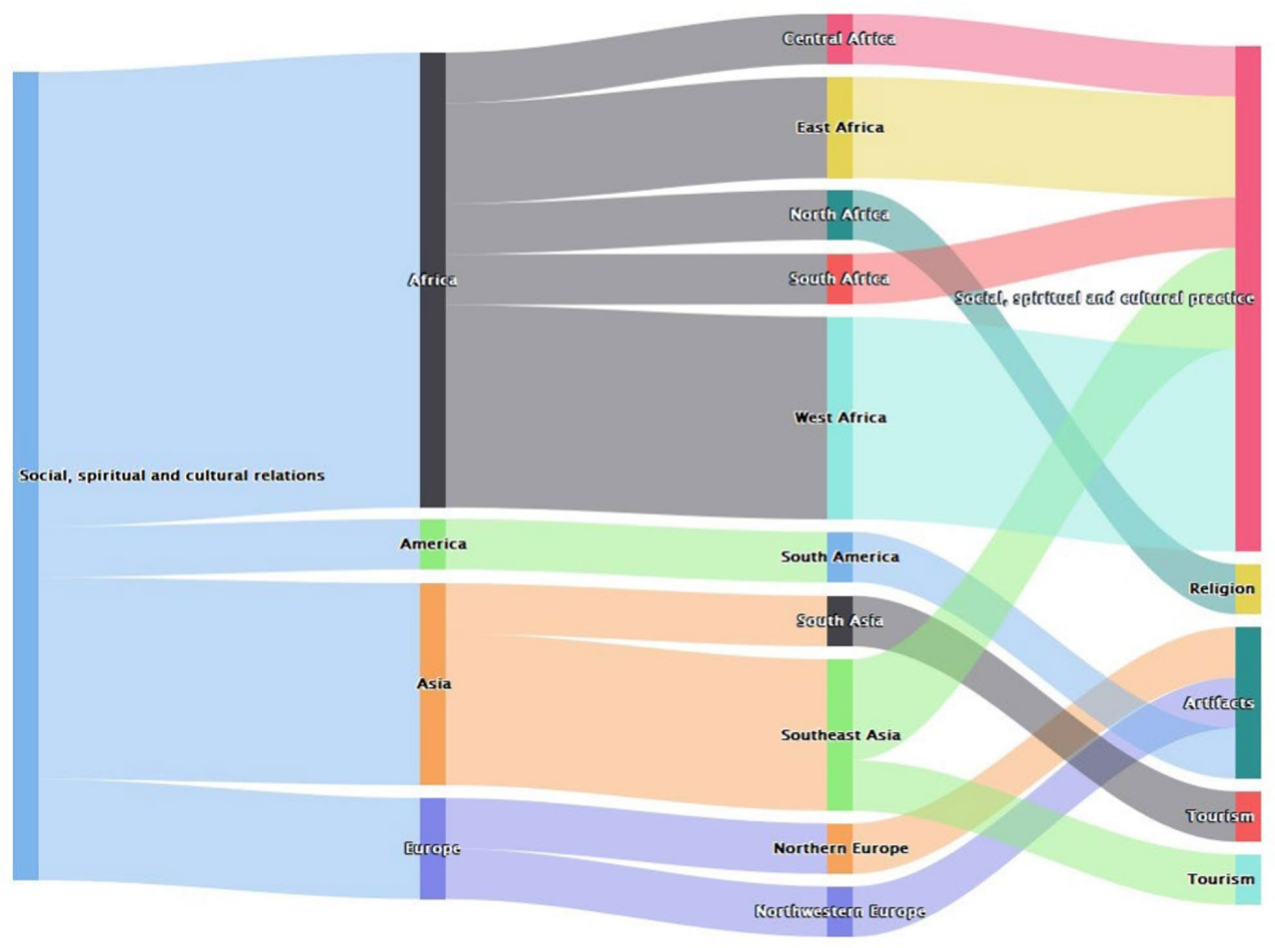
Geographical distribution of papers reporting social, spiritual and cultural benefits of bats globally (*n* = 16)

Within Africa, this category of benefits associated with bats is more pronounced in West Africa (Fig. 9). This could be explained partly by the differences in social organization across different communities [69]. Social organization within communities influences social capital and other capital assets that are critical in influencing the capability set [54], [107]. Moreover, it varies across space and time with differences in outcomes across the capability set [70].

Although literature is limited for social, spiritual and cultural relations benefits, bats have been noted to be critical in shaping how people conduct themselves as well as interact with each other. In African countries, bats influence spiritual relations which then influences how people undertake activities to ensure their livelihoods. For instance, traditional leaders in Ghana have been indicated to manage populations of bats and only permit hunting when necessary [71]. It is indicated that, when the population of bats has grown extremely high, the traditional leader would alert individuals to hunt for bats in sacred places [71]. This relationship with bats can have consequences on the livelihoods and well-being of the community members. Similar results have been registered in Nigeria where bats are considered as ghosts and usher peace to communities [61]. The bats have also been indicated to be vital in shaping social activities among community members. For instance, records in Guinea indicate bats to be hunted during the time of relaxation [72]. This kind of sport can be crucial as a livelihood component.

In Kenya, scientific literature has indicated the use of bats by witch doctors to cast problems among individuals [38]. Additionally, pastoralists in the southern Africa region including Namibia have been shown to attach rain onsets and offsets to bats [73]. This then has consequences on their grazing patterns and other activities. Meanwhile, in northern African countries like Egypt, bat symbols are drawn on artifacts indicating great value to ancient and contemporary cultures and religions [74].

Elsewhere in Asian countries like the Philippines, bats are associated with spirits that have consequences on human health [75]. They have also been used as charms in some communities while others have looked at them from a tourism perspective [75]. This then influences the activities that people pursue to ensure their livelihoods within such areas. Meanwhile, in Europe, the cultural benefits are scanty. However, bats have been drawn on artifacts as they are of great value to ancient and contemporary cultures and religions. This has been exemplified in Swedish cultures where bats are drawn on artifacts signifying sociocultural linkages among communities [76].

Bats also form an important part of totems among clan systems in some communities. This has been observed in Uganda and Cameroon where clan systems form an important part of the cultural systems [42]. This signifies a spiritual, religious, social and cultural association between human communities and bats [77]. Additionally, bats have been shown to improve relationships within communities. For instance, anecdotal records indicate bat fingers have been used culturally to enhance love potions among married partners in Uganda [41]. This perception is derived from the fact that bat fingers hold onto the tree tightly and such can be relevant in a relationship among partners. Besides the fingers, consumption of the head of the bats has been perceived to increase brightness of the children [41]. Although such data have not been published in scientific literature, it presents critical benefits that can fundamentally shape human livelihoods.

### Safety and security benefits associated with bats

Benefits of bats in respect to social, cultural and spiritual relations are more pronounced in Africa followed by Asia. In Africa, it is linked more with activities that are undertaken during social, spiritual and cultural relations. These activities are partly pursued in Asia as well as tourism. In Europe and America, bats activities were majorly pursued along the lines of artifacts.

The safety benefits associated with bats seem to be minimal. However, bats have been used as indicators of pollution as well as alerts for external threats. In African countries like Nigeria, bats have been used to alert communities of potential conflict threats [61]. This then allows for shielding among human communities.

## Discussion

The articles included in this review largely indicate wide-ranging socioeconomic benefits attached to bats. These benefits are highly variable across space and time. They cut across the human well-being components such as health, safety, material and immaterial assets, social, cultural and spiritual relations [78]. These benefits are pursued by human communities depending on the specific benefit expected. Health, material and immaterial benefits dominate among all other welfare components. In terms of spatial characteristics, these benefits attached to bats cut across the globe, but it is important to recognize the variation across regions (and countries) in terms of what is valued most. For instance, benefits of bats in terms of supporting human health are more pronounced in China than in other regions. This result is similar to what Tacket and colleagues reported with their review focused majorly on global medicinal benefits of bats [16]. Notably, they indicated more medicinal benefits of bats in Asia compared to other parts of the world. This variability can be explained by the fact that the way people pursue livelihoods is highly context dependent [15]. Notably, they highly vary across space and time. There are other factors that can influence the benefits that human communities derive from bats. These include existing social cultural practices, sources of livelihood, policies, among others. For instance, in China, traditional medicine has been integrated within the health care system [79], [114, 115]. This provides opportunities for the continued use of bat-derived products to address health challenges. Although the effectiveness of this value in human communities is unclear, it provides an opportunity for enhancing community-led bat conservation so as to not deplete bat populations but rather protect them as a valuable resource. Future studies could be undertaken to understand opportunities for capitalizing these aspects to support conservation of bat populations. In Africa, although the use of bats as medicine was expected to be higher than that in Asia, this study indicates otherwise. This may be associated with the low publication outputs within Africa. The higher medicinal benefits attached to bats is expected to be higher in Africa compared to Asia as reports indicate over 60% traditional medicine is used Africa [60]. Future studies thus ought to use primary approaches to understand the variation in the use of bats for medicinal purposes. Documenting such data will support actions for conservation of bat populations while protecting human health. Such primary data will be useful in addressing biases associated with variation in publication outputs across different regions.

Bats carry enormous socioeconomic value around the world as providing health benefits to people, yet several high consequence emerging infectious diseases have been associated with bats that may destabilize these traditional values. Disease agents such as Marburg virus, Nipah virus, coronaviruses spill over into human populations, creating a public health concern associated with bats rather than a beneficial outlook. Public fear of bats has triggered mass extermination events, such as in Kitaka mine, Uganda [80]. Yet, this issue also speaks to the need for a healthy balance between human and bat contact that protects human health as well as conserves bat populations for their continued material and ecological value. One Health interventions that contribute to this double win will thus be crucial.

This study indicated material and immaterial benefits attached to bats to be more pronounced and especially in Africa compared to other regions. This finding could be due to the fact that livelihood opportunities are limited in many parts of Africa [63]. This provides opportunities for continued exploitation of bats for a range of benefits including food, fertilizers, among others. However, this benefit with respect to bats is less compared to Africa. This can be explained by the fact that Asian countries have more robust economies that create opportunities for exploring diverse material and immaterial assets. This limits engagement in activities that focus majorly on bats for a livelihood. Although this review indicates this difference, future studies ought to explicitly explore using primary methods. Such studies should utilize welfare economic approaches to understand the driving factors behind differences in material and immaterial assets associated with bats. This will be a steppingstone for improved design of bat conservation interventions. Indeed, similar ideas have been urged by organizations focused on supporting conservation of bats. For instance, the Bat Conservation Trust has suggested ideas that include design of products with bat images [81]. These products can be sold and generate funds for improving conservation programs.

The use of bats for deriving health-related benefits was common across different countries in the globe. For instance, treatment of asthma using bats was reported in almost all the regions [65, 66, 81], [104]. Similar result has been reported in a review by Tacket and colleagues [16]. For many of the health complications, bats were used as a treatment probably due to limited access to formal health services. This has been observed in previous reviews, e.g., [16]. While the beliefs behind many of these benefits are not well documented, there is need for more studies to understand the reasons for use of bats and how they are utilized for these values. This has been by stressed in previous studies including that by Marco [60]. The treatment of asthma with bats has been well documented in the literature. Although this benefit is reported especially in Africa and Asia, there was no evidence that bats have any medicinal effect. While many studies did not specify the parts of the bats and the procedures utilized while addressing such ailments, this result reflects an important benefit that can be leveraged on to support conservation interventions. However, scientific analysis could be done to understand the medicinal effects of different parts of the bats. These analyses will support design of strategic conservation campaign programs while ensuring improved public health outcomes.

It has been noted that management of wildlife and ecological systems requires among others the advancement of human well-being [82]. This is because the sustainability of natural resources including bat populations cannot be addressed separately from the livelihoods of communities that rely on them for their welfare [83]. Therefore, to strike the balance, integrated approaches are needed to foster conservation of bat populations. Notably, equitable advancement of human well-being that does not compromise the health of bat populations would yield important benefits. This requires gaining people’s support and promoting their involvement in conservation of bats and their management. Such interventions could be aligned with people’s values attached to bats.

Beyond the pace of new studies being published outside our search timeframe, our review was subject to a number of limitations. We only utilized English language in undertaking the literature review process. While we did not add studies written in other languages, focusing on reference list of extracted papers and including other studies may highlight an important observation of research on values of bats. Our approach did not explicitly include ecological values associated with bats as well as whether the benefits attached increased risk of disruption of bat populations. The link between benefits that human communities derive from bats as well as implications on their populations is widely recognized but fell beyond the scope of this review. Finally, our review was limited to the positive benefits that human communities associate with bats. It is certain that many of negative benefits associated with bats are also vital in designing conservation actions but were not included in our review.

## Conclusion

Bat populations are known to be declining at a rapid, but heterogeneous pace across most countries and regions globally. A growing body of literature is available to better understand the benefits of bats and how they could be used to support their conservation. However, in the past years, these have not been fully utilized to enhance campaigns for conservation of bat populations. Most research focuses on health, material and immaterial assets, while benefits along the line of safety, sociocultural and spiritual aspects may have been overlooked over time. Finally, future research efforts should prioritize use of primary research approaches to better understand socioeconomic benefits of human well-being. Additionally, such studies should focus on designing methodologies that allow for comparison of results as well as support policy decisions. Finally, while our analysis only elucidated ecosystem services only within the category of material and immaterial assets, the results may not account for all this type of benefits. There is thus a need for pragmatic approaches that combine socioeconomic and ecosystem benefits of bats which ought to be developed.

### Supplementary Information

Supplementary Material 1.

Supplementary Material 2.

## Data Availability

Data for this work has been submitted as part of supplementary documents. The code for analysis is available at https://github.com/SiyaAggrey/socioeconomic-values-of-bats.

## Abbreviations

DNA: Deoxyribonucleic acid
UISTF: Uganda Independence Scholarship Trust Fund
